# Dangers of Oratory

**Published:** 1876-02

**Authors:** 


### DANGERS OF ORATORY.

AMONG the greatest orators of the last hundred years, there are many who have gone down to a drunkard's degradation and drunkard's grave. Into the kennel, men of might have gone from the editorial sanctum, from the halls of legislation, from the forum, from the bar, from the stage, and from the pulpit itself, as witness—but we spare the dead, in fact, or in effect—any man of extensive observation can count up for himself. We can make use of the lesson, without harrowing up the feelings of the living.

This terrible vice of drunkenness is fallen into by the slowest degrees, by that class of young men who are considered "good at making a speech." Such soft names are sometimes given to liquor-drinking, that the designation itself is a lure—it at least is not a scare-crow. "To steady the nerves," "to wet the whistle," and the like.

Some persons "take a drink" before making a speech; others, during the time; a third class prefer taking it afterwards, which is certainly the safest. One of the greatest of modern orators arose to address an assembly in this city, in our hearing, but was so drunk that he not only had not the full command of his tongue and lips, but had to hold up himself by the support of the corner of a table. He had miscalculated!—not as to the quantity of liquor, or as to the time it would take to begin to affect him—he had doubtless made the calculation so often, and had gone through the "operation" so repeatedly, as to feel perfectly at home; the error was in his predecessor being too dull, and, as a consequence, too long, and the great man missed fire. EDMUND KEAN, the great player, never drank during a performance, but *always* after it, and drink killed him, before his time. KNOX, a celebrated English actor, required two quarts of brandy to enable him to go through, properly, with one of his great characters. And it was said of big JOHN REEVE, that he required a still greater quantity.

It is always safest and best for a man to rely on his own resources. He may not be so great "on occasion," but he will be more uniformly great, without the risk of disgracing himself and putting his friends and admirers to the blush.

